# The lived experience of adults with cystic fibrosis: what they would tell their younger selves about the gut

**DOI:** 10.1111/jhn.12703

**Published:** 2019-11-25

**Authors:** L. Cave, L. J. Milnes

**Affiliations:** ^1^ Children's Nutrition and Dietetics Leeds Teaching Hospitals NHS Trust Leeds UK; ^2^ School of Healthcare University of Leeds Leeds UK

**Keywords:** cystic fibrosis, gut, lived experience, patient perspective, self‐management

## Abstract

**Background:**

Little is known about adults’ experience of living with cystic fibrosis (CF) specifically in relation to the gut. However, their unique perspectives may be meaningful to children with CF and inform the understanding and practice of dietitians. The present study aimed to explore adults’ lived experience of the CF gut and how they learnt to manage the gut as they were growing up.

**Methods:**

Semi‐structured interviews were conducted with adult inpatients (*n* = 10). Interviews were audio‐recorded, transcribed verbatim and accounts analysed using interpretative phenomenological analysis.

**Results:**

Three super‐ordinate themes were identified: *taking Creon*,* the learning process* and *this much I (now) know*. Participants accounts of how CF affects the gut predominantly focused on taking Creon (pancreatin, Mylan). Various strategies were employed for coping with peer responses to taking Creon at school. Several participants reached adulthood before they understood and/or accepted that taking Creon consistently needed to be normal for them. Knowledge and understanding developed over time, with ‘CF experience’ and was shaped by family, CF care teams and other children with CF. All had unmet information needs when growing up. Having key explanations earlier, to make connections between eating, taking Creon, gaining weight and growth, did or would have helped most participants. Participants urged children to be assertive, ask questions and not only be involved in managing their diet and gut, but also begin to take control of this aspect of their CF.

**Conclusions:**

Supporting development of knowledge, skills and confidence to manage diet and the gut needs to be integral to care throughout childhood.

## Introduction

Cystic fibrosis (CF) is a multisystem condition that predominantly affects the respiratory and gastrointestinal (GI) systems 1. In recent years, the focus in clinical practice has been on managing complications of the respiratory system 2  as respiratory failure secondary to progressive lung disease is the most common cause of death 3. However, there are now calls to redress the balance and consider the non‐respiratory manifestations of CF, in particular, the GI system (or ‘gut’) 4, with relief of GI symptoms identified as a research priority for CF 5. This is because effective management of the gut, through control of symptoms and optimising nutritional status, has a major impact on quality of life 6 and survival 7.

Previous research has explored adults experience of living with CF 8, 9, 10, 11, 12, although not specifically in relation to the gut. Where this has been carried out in other chronic GI‐related conditions, patients’ perspectives have helped inform clinicians understanding and practice 13, 14, 15.

The present study therefore aimed to explore adults lived experience of the CF gut, how they made sense of their experiences and how they learnt to manage the gut as they were growing up. Their unique insights will be shared with children with CF. This is because young people have previously identified information from peers with the same long‐term condition to be particularly relevant to them and their experiences 16. Through this specialist type of peer support, children may feel connected and better supported to make choices around self‐care 17. This is important  as children begin to gain independence, for example, in making food choices and adjustments to their accompanying pancreatic enzyme replacement therapy (PERT). The study findings will also inform dietitians understanding of patient needs and how patient‐centred care can be enhanced 18 with children with CF and their families.

## Materials and methods

### Design

This qualitative study adopted the approach of interpretative phenomenological analysis (IPA) because this was appropriate for gathering in‐depth accounts of individuals lived experiences and exploring the meanings they attribute to these experiences 19.

The study design, method and setting for data collection were chosen after consultation with four patients attending a single regional adult CF centre; in addition, one patient reviewed and provided valuable feedback on all study documentation (all nonparticipants).

Ethical approval was granted by South West‐Exeter Research Ethics Committee, reference: 17/SW/0042.

### Sampling and recruitment

Purposive sampling was conducted at a single regional adult CF centre in the UK. A sample size of 8–10 potential participants was aimed for because IPA studies are concerned with a concentrated focus on a small number of individuals, typically less than 10 19.

The lead dietitian in the direct clinical care team identified eligible participants from electronic medical records based on the criteria in Table 1.

**Table 1:** Eligibility criteria for potential participants

Eligibility	Rationale
Inclusion	
Adults diagnosed with cystic fibrosis (CF) in early childhood	Adults asked to reflect on their experience of growing up with CF
Aged 20–30 years	Upper age limit of 30 years so they all had experience of the same ‘high fat, high calorie CF diet’ and used the same preparation of PERT that is currently available to children; lower age limit of 20 years so they were out of their teens
Pancreatic insufficient (PI), so required pancreatic enzyme replacement therapy (PERT)	To be able to explore their experience of managing PERT, how this changed over time and how they developed their knowledge and understanding
	(Approximately 85%–90% of people with CF are PI. In clinical practice, the majority of children find it challenging to manage PERT, particularly as they move into adolescence)
Admission for routine intravenous antibiotic therapy (IVAT) between May and July 2017	This was the preferred setting chosen by patients over the 3‐month period for data collection
Exclusion	
Approximately continuous IVAT over the previous year	Severely affected by CF and possibly too unwell to participate
On the active transplant list	
Pregnant	Already committing extra time for close monitoring

The lead dietitian approached and invited potential participants to take part in the study when they were part way through an admission and were clinically stable. All were provided with written study information and the opportunity to ask questions. Written informed consent was obtained by the first investigator immediately prior to participation.

### Data collection

Ten potential participants were approached and all agreed to participate (five females and five males; aged 19–30 years) (Table 2). Single one‐to‐one semi‐structured interviews were conducted by the first investigator throughout May and June 2017. Interviews were up to 1 h in duration and conducted in patients own rooms on the ward, with only the participant and the researcher present. A topic guide (Table 3) was used flexibly. Interviews were audio‐recorded, transcribed verbatim and transcripts annotated with field notes made after each interview. The first two interviews enabled pilot testing of the topic guide; these two interviews were included in the final analysis, as no changes were required. Where possible, data were collected and analysed concurrently.

**Table 2:** Characteristics of participants (*n* = 10)

Pseudonym	Gender	Age (years)	Age at CF diagnosis
Anne	F	30	3 months
Christine	F	21	4 months
David	M	28	1 month
Emma	F	19	1 month
Jane	F	27	At birth
John	M	24	13 months
Mark	M	27	At birth
Philip	M	27	2 years
Steven	M	24	3 weeks
Susan	F	28	18 months

CF, Cystic fibrosis; F, female; M, male.

**Table 3:** Interview topic guide

Topics for discussion	Experience of CF and gut involvement for them
	Experience as a child of eating, taking Creon, managing their gut (prompts: experience at school, with friends, on trips; coping strategies)
	How experiences have changed over time (insights of their journey) (prompts: attitudes, knowledge, understanding, behaviours, motivation)
	Facilitators
	Something or someone that particularly helped them make sense of how CF affects their gut
	(any lightbulb moment/specific event that made things click in terms of their understanding)
	(prompts: any appropriate, relevant support and guidance received – from HCPs/family/friends (with or without CF)
	Barriers
	Anything they wish they were told as a child – if so, when, how and by whom
	Insights/explanations they feel would be important to share with a child with CF (‘top tips’ to pass on)
	Anything else they would like to add

CF, cystic fibrosis; HCP, healthcare professional.

### Data analysis

Anonymised transcripts were analysed systematically using IPA, following the process described by Smith *et al*. 19 In summary, a transcript was read and re‐read and exploratory comments added to describe content, use of language and concepts. Connections and patterns between exploratory comments were mapped to identify emergent themes and these clustered to form themes. This process was then repeated for each transcript and patterns identified across the sample as a whole. Themes derived from the data (LC) were independently checked (LM) and a high degree of agreement taken as validation of identified themes.

## Results

Three themes were inferred: taking Creon (pancreatin, Mylan), the learning process and ‘this much I (now) know’. Within these themes, several subthemes emerged, each supported by exemplar quotations from participants. These are presented in the narrative below and in Table 4.

**Table 4:** Themes and subthemes supported by exemplar participant quotations

Theme and subthemes	Participant quotations
Taking Creon	
Coping with peer responses	[At primary school] ‘It was just realising you’re a bit different from all the other kids. In secondary school, obviously that’s when they did ask more questions, they understood then, ‘well we don’t have to take medicines when we eat, so why do you?’ (Christine) [At secondary school] ‘A couple of people clocked me taking my tablets (Creon) and I started getting bullied. So, I kind of stopped taking them and that upset my stomach a lot, but I didn’t care I just sort of suffered it out through the daytimes at school’ (Susan)
‘This is our normal’	‘It was just a normal part of my life to take Creon. From quite an early age I was trusted to do it’ (Jane) ‘Everyone’s different. It’s finding out what you need, what works best for you and just always remembering to have it’ (Steven)
The learning process	
Family	‘In my family… I didn’t grow up with routine… whenever I went in [for a hospital admission] it was brilliant ‘cos I got up to date with all my meds, I got used to everything’ (David) ‘The dietitians were telling my mum what foods are high in fat and then I learnt from her… I relied on my mum a lot to know how many (Creon) I needed to have’ (Christine)
CF team	‘If you’ve got questions – just ask – don’t sit there quietly thinking you don’t want to ask because you don’t want to get into trouble for not knowing the answer; that’s what I did, I always wondered about stuff and then I was never asked’ (Anne) ‘I moved to X (a specialist CF centre) and it was completely different to what I was used to. I felt more involved, definitely. Rather than them telling me stuff, they were asking me stuff and including me with decisions’ (Steven)
Other children with CF	[Children with CF now segregated] ‘They’ll have no‐one really that they can relate to… they may want some reassurance that they’re not the only one going through it and ask questions on how you deal with things’ (Susan) ‘Cross‐infection wasn’t in then… (during admissions) there was a lot of CFs and diabetics together – we had takeaway nights and could talk and ask each other questions’ (Anne)
‘This much I (now) know’	
Knowing things earlier	‘I had it explained and explained to me (why I needed to take Creon) but I never listened. I did know about it, I just pretended not to, just silly really’ (Philip)
Making connections	‘I was always told I need to keep gaining weight to make me grow, and ‘cos I wanted to be tall, that always made me motivated to eat more… and it was always said eating gives you energy and you’ll be able to do more and ‘cos I love doing sport, it was always important for me to eat’ (Emma) ‘If I knew taking Creon contributed to growth, I’d probably have taken it more, as I would have liked to be taller… but not knowing that, I just thought it (Creon) was something and nothing, so I just shrugged them off’ (John)
Thinking longer‐term	‘Explain what’s happening ‐ for the long‐term benefits it’ll bring – that it’s keeping them well’ (Mark) [To a child with CF] ‘Get involved – be in control ‐ do what you need to do and you’ve got a bright future ahead of you’ (David)

CF, cystic fibrosis.

### Taking Creon

Participants’ accounts of how CF affects the gut, predominantly focussed on taking Creon (the most widely available brand of PERT in the UK). A wide range of views were expressed about the continual need for Creon and the tension this could create.

#### Coping with peer responses

Taking Creon was described as the most visible part of having CF when at school. Peer responses were on a spectrum, from bullying and accusations of being a drug addict, through to friends giving reminders to take it. Peers at primary school were generally accepting but, at secondary school, they more readily recognised difference and questioned it. Various coping strategies were adopted, including not taking Creon at all, taking it out of sight of others, not eating or choosing foods that required less or no Creon. Although some participants felt embarrassed about getting their Creon ‘out’, others cautioned against hiding it and advised either ignoring what peers say, or giving a simple explanation to close down further questioning.

‘If anyone asked, I explained why it was needed. I'd just tell them they are enzymes and you make them in your body but I don't, so I just need to have them when I have some food so my body can break down the food and absorb all the nutrients out of it’ (Mark)

This coping strategy relied on them having an explanation they understood and felt confident to share, though as adults this strategy was reported as the most widely adopted.

#### ‘This is our normal’

All participants reported that as they grew older and the course of CF progressed, they realised how important Creon was and accepted that taking it was a normal part of life for them. For several, this realisation came as a result of severe weight loss and they described almost giving up fighting against it.

‘Cos obviously I wasn't taking them (Creon) my food wasn't digesting or anything, so I was just losing weight, losing weight. I ended up, it got that bad I had to have a feeding tube put in and that's when I realised, it'd be better just to take them’ (Philip)

For some, it was only when they started taking Creon that they realised what it did because symptoms of malabsorption were normal for them. Many reported that it was a process of trial and error to find the doses and timing of Creon that suited their individual bodies. Without exception, all participants stressed that taking Creon consistently is essential and offered encouragement to children to build this in as normal.

### The learning process

All participants shared the view that they had developed their knowledge and understanding as a gradual accumulation of learning over time, with ‘CF experience’.

#### Family

Family support ranged from taking complete control through to participants having to deal with CF on their own from an early age. Where parents took control and did everything for them, this appeared to hinder development of their own understanding. Several participants described intermittent or erratic family involvement and felt they did better when they were an inpatient. A lack of explanations from their families left them unaware of why treatments were needed and this affected adherence, particularly to Creon. Others reported having supportive families, where it appeared that they were dealing with it together and information was shared about how to manage diet and Creon.

‘The way my family approached it … I never felt it was an illness when I was younger, it was always just “ah this is what I need to do”, just everyday’ (Emma)

All participants expressed how the type of family support they received shaped their understanding and confidence. Participants urged parents of children with CF to provide time, space and encouragement to develop independence,  whilst also providing a safety net.

#### Cystic fibrosis team

Most participants did not feel included at clinic visits when they were a child and urged CF teams to do more to directly engage and involve children. Several described only the adults speaking and they were left feeling ‘why am I here?’.

‘Children's clinic was always waiting round a lot. They'd obviously do whatever checks they needed to and then it would just be them talking to my mum. I was just always waiting to leave instead of them talking to me’ (John)

There were exceptions, particularly if they attended a specialist paediatric CF centre, with some reporting their CF team an asset in informing their learning. Participants acknowledged the need for children to be assertive, to actively seek support and ask questions. Some expressed regret that they had not done so themselves and urged children to be braver than they had been.

#### Other children with cystic fibrosis

Participants described how other children with CF were key contributors to their learning. All acknowledged that there was no strict segregation (to reduce the risk of cross‐infection) when they were children and several expressed concern that children now will be even more isolated than they were.

### This much I (now) know

All participants reflected that as children growing up with CF, they had some unmet information needs. This varied from needing information on everything, through to wanting explanations on why specific vitamins were needed.

#### Knowing things earlier

Most participants felt it would have made a difference to have had things explained earlier, in terms they could understand, with things revisited at regular intervals and in greater depth as they grew older. They described how this would have given them more confidence in what was happening.

‘I never got it explained so I grew up thinking “well, I don't actually know why I'm doing this, so I might as well not do it” … I wish I was told everything, appropriate for the age you are, in language you can understand and then as you grow up, just that little bit more information, just gradually’ (Susan)

However, some acknowledged they were not or would not have been ready to take on the information. Only a few felt they got the explanations they needed at an appropriate level and age; though as children, these participants were confident to ask questions and felt supported within their family units.

#### Making connections

Most participants described themselves as scrawny or skinny children, some small in stature, who on the whole, had large appetites and good lung function. Some reported having the information they needed to make necessary connections between eating, taking Creon, gaining weight and growing. Several highlighted how it had been helpful to link eating well with having energy to be active (e.g. to play out with friends and play sport).

‘Food plus Creon equals weight … means less admissions and more time with your friends’ (David)

Others felt that having this information would have helped them to make sense of things and given them an incentive to eat more and improve their Creon adherence. Several participants had not made the connection between weight and lung function until they were adults. Some felt it would have been helpful to know this earlier, whereas others felt it would have put even more pressure on eating and gaining weight and so were glad to have not known sooner.

#### Thinking longer‐term

Although some participants described embracing a high‐energy, high‐fat ‘CF diet’ and having no wish to change this, several described how their diets had changed over time to become more balanced, as a result of thinking more about their long‐term health, not just their CF.

‘I always remember dietitians saying about calories and needing to eat more fat … “cos people didn't live as long it didn't matter … but now it should be about how important nutrition is generally, for your health as a whole’ (Jane)

These participants expressed how children with CF need to be motivated to begin looking after their diet and gut in ways that relate positively to the future and goals for their adulthood.

## Discussion

Through in‐depth interviews, adults talked at length about their lived experiences of CF and what had shaped their knowledge and understanding of the CF gut. They were enthusiastic about sharing their insights with children with CF and optimistic about children's futures. They suggested how families and CF teams could promote children's learning and expressed concern at the current lack of peer support.

As reported previously 11, 20, 21, various coping strategies were adopted for taking Creon, (particularly at secondary school), to reduce feelings of difference from their non‐CF peers. However, participants advised children with CF to not hide their Creon but, instead, to offer a simple explanation to peers as to why it is needed. This has implications for practice as children clearly need support in preparing for transition to secondary school not only in increasing their independent Creon taking, but also in being equipped with the knowledge, skills and confidence to relay explanations to peers.

Knowledge and understanding developed gradually as an accumulation of learning over time, as described previously 8. Families, the CF team and peers with CF played important roles in informing participants learning. The role of families in passing on information about diet and Creon was essential to their understanding and confidence and impacted on their Creon adherence. The link between family functioning and treatment adherence in CF has been identified previously 22, 23, 24, 25, with families that find a balance between cohesion and flexibility reporting higher rates of adherence 22. In the present study, participants in supportive family units were, as children, confident to ask questions and seek the explanations they needed from the CF team; however, all participants urged children to be assertive, seek support and be actively involved in their dietary care.

Advice to the CF team strongly resonated with that highlighted previously 26, 27, with a call for a more child‐centred approach to dietary consultations, where children can actively participate rather than feel marginalised, and discussions use terms relevant and meaningful to children's daily lives.

Interaction with other children with CF made a significant contribution to participants learning, supported their interactions with the CF team and reduced feelings of difference and isolation. This was congruent with findings from other studies 21, 28, 29. With strict segregation now in force, virtual peer interaction may play an important role 30, 31, although creating online platforms suitable for primary school age children remains a challenge.

As reported previously 32, all participants had some unmet information needs when growing up. As a consequence, several described a decline in adherence during adolescence. This may impact on health outcomes such as growth and lung function 33 and contribute to reduced survival in adulthood. Information needs must therefore be addressed throughout childhood as part of developing a solid grounding in self‐management skills to take forward into adolescence 34, 35.

### Study strengths and limitations

All participants attended the same single regional adult CF centre. However, they shared experiences of attending different regional and shared‐care paediatric CF centres across the UK and were therefore exposed to different service provision when growing up with CF.

The present study focussed only on adults who were pancreatic insufficient. The experiences of adults who are pancreatic sufficient warrant full consideration in their own right and could be the focus of a future study.

Participants may have responded differently if interviewed in a different setting. Although they appeared relaxed in their own rooms on the ward, the study captured how they made sense of their experiences at one point in time, in the context of a single one‐off interview. The data generated were a co‐production between participant and researcher 36 and the findings are an interpretation of participants’ accounts by LC and checked by LM. There was insufficient time for participants to feedback on the derivation and identification of themes; however, a journal was kept throughout by LC to reflect on how prior knowledge and experience as a children's CF dietitian may impact on the data.

## Conclusions

Supporting children with CF to develop their knowledge, skills and confidence to manage diet and the gut needs to be integral to care throughout childhood. Targeting this support in preadolescence may equip children to effectively manage the demands of this aspect of CF care during adolescence. The findings of the present study inform a current programme of work identifying how this support can be delivered as part of routine dietetic practice and how families, CF teams and peer support may enhance this.

## Conflicts of interests, source of funding and authorship

The authors declare that they have no conflicts of interest.

Laurie Cave was funded by a National Institute for Health Research (NIHR) Masters in Clinical Research Studentship for this research project. This publication presents independent research. The views expressed are those of the authors and not necessarily those of the NHS, the NIHR or the Department of Health and Social Care.

LC and LM were responsible for the study design. LC performed the data collection. LC and LM performed the data analysis. LC and LM prepared the draft of the manuscript. LM supervised the study. All authors critically reviewed the manuscript and approved the final version submitted for publication.

## Transparency declaration

The lead author affirms that this manuscript is an honest, accurate and transparent account of the study being reported. The reporting of this work is compliant with COREQ guidelines 37. The lead author affirms that no important aspects of the study have been omitted and that any discrepancies from the study as planned have been explained. The dataset and study documentation are available at: https://doi.org/10.5518/609.

## References

[1] Bishay LC & Sawicki GS (2016) Strategies to optimize treatment adherence in adolescent patients with cystic fibrosis. Adolesc Health Med Ther 7, 117–124. 27799838 10.2147/AHMT.S95637 PMC5085292

[2] Freedman SD & Schwarzenberg SJ (2016) Beyond the lungs: the new GI frontier. J Cyst Fibros 15, 409–410. 27264963 10.1016/j.jcf.2016.05.008

[3] Elborn SJ (2016) Cystic fibrosis. Lancet 388, 2519–2531. 27140670 10.1016/S0140-6736(16)00576-6

[4] Cystic Fibrosis Trust (2016). News of the Trust's latest research. Available at: https://www.cysticfibrosis.org.uk/news/chewing-the-fat (accessed June 2017).

[5] Rowbotham NJ , Smith S , Leighton PA *et al* (2018) The top 10 research priorities in cystic fibrosis developed by a partnership between people with CF and healthcare providers. Thorax 73, 388–390. 28778919 10.1136/thoraxjnl-2017-210473 PMC5870449

[6] Shoff SM , Tluczek A , Laxova A *et al* (2013) Nutritional status is associated with health‐related quality of life in children with cystic fibrosis aged 9‐19 years. J Cyst Fibros 12, 746–753. 23410621 10.1016/j.jcf.2013.01.006 PMC3683079

[7] Yen EH , Quinton H & Borowitz D (2013) Better nutritional status in early childhood is associated with improved clinical outcomes and survival in patients with cystic fibrosis. J Pediatr 162, 530–535. 23062247 10.1016/j.jpeds.2012.08.040

[8] Huyard C (2008) Exploring one's own human condition: adults affected by cystic fibrosis. Qual Health Res 20, 535–544. 10.1177/1049732308315108 18354051

[9] Badlan K (2006) Young people living with cystic fibrosis: an insight into their subjective experience. Health Soc Care Community 14, 264–270. 16650123 10.1111/j.1365-2524.2006.00619.x

[10] Gjengedal E , Rustoen T , Wahl AK *et al* (2003) Growing up and living with cystic fibrosis: everyday life and encounters with the health care and social services – a qualitative study. Adv Nurs Sci 26, 149–159. 10.1097/00012272-200304000-00007 12795543

[11] Lowton K & Gabe J (2003) Life on a slippery slope: perceptions of health in adults with cystic fibrosis. Sociol Health Illn 25, 289–319. 14498923 10.1111/1467-9566.00348

[12] Tracy JP (1997) Growing up with chronic illness: the experience of growing up with cystic fibrosis. Holist Nurs Pract 12, 27–35. 9384068 10.1097/00004650-199710000-00006

[13] Sammut J , Scerri J & Xuereb RB (2015) The lived experience of adults with ulcerative colitis. J Clin Nurs 24, 2659–2667. 26111098 10.1111/jocn.12892

[14] McCormick JB , Hammer RR , Farrell RM *et al* (2012) Experiences of patients with chronic gastrointestinal conditions: in their own words. Health Qual Life Outcomes 10, 25. 22401607 10.1186/1477-7525-10-25 PMC3349594

[15] Fletcher PC , Schneider MA , Van Ravenswaay V *et al* (2008) I am doing the best that I can!: Living with inflammatory bowel disease and/or irritable bowel syndrome (part II). Clin Nurse Spec 22, 278–285. 18955845 10.1097/01.NUR.0000325382.99717.ac

[16] Milnes LJ , McGowan L , Campbell M *et al* (2013) A qualitative evaluation of a pre‐consultation guide intended to promote the participation of young people in asthma review consultations. Patient Educ Couns 91, 91–96. 23142213 10.1016/j.pec.2012.10.010

[17] National Endowment for Science, Technology and the Arts (NESTA) . (2013). People helping people: peer support that changes lives. Available at: https://www.nesta.org.uk/report/people-helping-people-peer-support-that-changes-lives/ (accessed July 2019).

[18] Sladdin I , Chaboyer W & Ball L (2018) Patients’ perceptions and experiences of patient‐centred care in dietetic consultations. J Hum Nutr Diet 31, 188–196. 28891086 10.1111/jhn.12507

[19] Smith JA , Flowers P & Larkin M (2009) Interpretative Phenomenological Analysis: theory, Method and Research. London: SAGE.

[20] Jessup M & Parkinson C (2010) ‘All at sea’: the experience of living with cystic fibrosis. Qual Health Res 20, 352–364. 19955225 10.1177/1049732309354277

[21] Christian BJ & D'Auria JP (1997) The child's eye: memories of growing up with cystic fibrosis. J Pediatr Nurs 12, 3–12. 9037958 10.1016/S0882-5963(97)80016-4

[22] White T , Miller J , Smith GL *et al* (2009) Adherence and psychopathology in children and adolescents with cystic fibrosis. Eur Child Adolesc Psychiatry 18, 96–104. 18807223 10.1007/s00787-008-0709-5

[23] Everhart RS , Fiese BH , Smyth JM *et al* (2008) Family functioning and treatment adherence in children and adolescents with cystic fibrosis. Pediatr Allergy Immunol Pulmonol 27, 82–86. 10.1089/ped.2014.0327 PMC4062112 24963456

[24] Berge JM & Patterson JM (2004) Cystic fibrosis and the family: a review and critique of the literature. Fam Syst Health 22, 74–100.

[25] DeLambo KE , Levers‐Landis CE , Drotar D *et al* (2004) Association of observed family relationship quality and problem‐solving skills with treatment adherence in older children and adolescents with cystic fibrosis. J Pediatr Psychol 29, 343–353. 15187173 10.1093/jpepsy/jsh038

[26] Savage E & Callery P (2007) Clinic consultations with children and parents on the dietary management of cystic fibrosis. Soc Sci Med 64, 363–374. 17064831 10.1016/j.socscimed.2006.09.003

[27] Savage E & Callery P (2005) Weight and energy: parents’ and children's perspectives on managing cystic fibrosis diet. Arch Dis Child 90, 249–252. 15723908 10.1136/adc.2004.056804 PMC1720287

[28] Tierney S , Deaton C , Jones A *et al* (2013) Liminality and transfer to adult services: a qualitative investigation involving young people with cystic fibrosis. Int J Nurs Stud 50, 738–746. 22624956 10.1016/j.ijnurstu.2012.04.014

[29] Berge JM , Patterson JM , Goetz D *et al* (2007) Gender differences in young adults’ perceptions of living with cystic fibrosis during the transition to adulthood: a qualitative investigation. Fam Syst Health 25, 190–203.

[30] Moola F (2018) The complexities of contagion: the experience of social isolation among children and youth living with cystic fibrosis in Canada. J Child Health Care 22, 4. 10.1177/1367493518767784 29614879

[31] Kirk S & Milnes L (2016) An exploration of how young people and parents use online support in the context of living with cystic fibrosis. Health Expect 19, 309–321. 25691209 10.1111/hex.12352 PMC5055278

[32] Sawicki GS , Sellers DE , McGuffie K *et al* (2007) Adults with cystic fibrosis report important and unmet needs for disease information. J Cyst Fibros 6, 411–416. 17452026 10.1016/j.jcf.2007.03.004

[33] Eakin MN , Bilderback A , Boyle MP *et al* (2011) Longitudinal association between medication adherence and lung health in people with cystic fibrosis. J Cyst Fibros 10, 258–264. 21458391 10.1016/j.jcf.2011.03.005 PMC3114200

[34] Christian BJ & D'Auria JP (2006) Building life skills for children with cystic fibrosis: effectiveness of an intervention. Nurs Res 55, 300–307. 16980830 10.1097/00006199-200609000-00002

[35] Downs JA , Roberts CM , Blackmore AM *et al* (2006) Benefits of an education programme on the self‐management of aerosol and airway clearance treatments for children with cystic fibrosis. Chron Respir Dis 3, 19–27. 16509174 10.1191/1479972306cd100oa

[36] Mason J (2002) Qualitative Researching, 2nd edn London: SAGE.

[37] Tong A , Sainsbury P & Craig J (2007) Consolidated criteria for reporting qualitative research (COREQ): a 32‐item checklist for interviews and focus groups. Int J Qual Health Care 19, 349–357. 17872937 10.1093/intqhc/mzm042

